# Acute Effects of Combining Whole-Body Electromyostimulation with Resistance Training in Active Women

**DOI:** 10.3390/jfmk9010010

**Published:** 2023-12-29

**Authors:** Andrea Buonsenso, Marco Centorbi, Giulia Di Martino, Carlo Della Valle, Gloria Di Claudio, Domenico Di Fonza, Erika Di Zazzo, Giuseppe Calcagno, Alessandra di Cagno, Giovanni Fiorilli

**Affiliations:** 1 Department of Medicine and Health Sciences, University of Molise, 86100 Campobasso, Italy; andreabuonsenso@gmail.com (A.B.); marco.centorbi@hotmail.it (M.C.); giulia.dimartino21@gmail.com (G.D.M.); carlodv95@gmail.com (C.D.V.); diclaudiogloria@gmail.com (G.D.C.); d.difonza@studenti.unimol.it (D.D.F.); erika.dizazzo@unimol.it (E.D.Z.); giuseppe.calcagno@unimol.it (G.C.); fiorilli@unimol.it (G.F.); 2 Department of Neuroscience, Biomedicine, and Movement, University of Verona, 37124 Verona, Italy; 3 Department of Movement, Human and Health Sciences, University of Rome “Foro Italico”, 00135 Rome, Italy

**Keywords:** whole body electromyostimulation, isometric strength, active woman, CK levels

## Abstract

Strength training elicits benefits both in performance and on a psychological level in women, such as increased muscle strength and improved self-esteem. Whole-body electromyostimulation (WB-EMS) could be a training strategy for enhancing muscular strength. The aim of this study was to assess the acute effects of a single session of WB-EMS superimposed over classic resistance training on isometric strength, endurance strength and flexibility. Furthermore, the safety of the protocol was assessed by monitoring the levels of creatine kinase (CK) 48 h after the training protocol was completed. Sixteen active women (aged 22.06 ± 1.88) were randomly assigned to an experimental group (EG) (*n* = 8) and a control group (CG) (*n* = 8). The EG performed four sets of 12 repetitions of three strength exercises with superimposed WB-EMS, while the CG performed the same protocol without WB-EMS. RM-ANOVA showed a significant time*group interaction on posterior kinetic chain extensors’ mean and peak strength in the EG (F_(1,14)_ = 10.036; *p* = 0.007; and F_(1,14)_ = 20.719; *p* < 0.001; respectively). A significant time*group interaction was found in the sit and reach test for the EG (F_(1,14)_ = 10.362; *p* = 0.006). Finally, ANOVA performed on the CK levels showed no significant difference between the groups (F_(1,14)_ = 0.715; *p* = 0.412). WB-EMS training led to an immediate improvement in strength performance and flexibility, and this protocol was shown to be safe in terms of CK levels, 48 h after completing the training protocol.

## 1. Introduction

Involving women in physical activities is crucial for their health and to mitigate the risk of insufficient daily activity [1]. Specifically, strength training enables women to gain benefits, not only enhancing their quality of life but also extending their life expectancy [2]. Strength training brings various physiological benefits especially to women, such as increased muscle strength [3] and lean mass [4], reduced fat mass [4] and prevention of the loss of bone mineral density [5] and cardiovascular disease [6]. Furthermore, strength training yields several psychological benefits, including improved self-esteem [7], emotional well-being [8] and body image [8] and reduced state anxiety [9]. 

Whole-body electromyostimulation (WB-EMS) could be a different training strategy for enhancing muscular strength with different motor demands. WB-EMS induces a global-body electrical myostimulation, synchronously activating up to 8–10 different muscle groups (biceps, triceps, abdominal muscles, pectoralis major, upper and lower back, gluteus, quadriceps and hamstrings) while the subject performs functional movements during the stimulation. WB-EMS was demonstrated to activate high-threshold motor units [10] and improve motor nerve innervation, enhancing intramuscular coordination [11]. Additionally, it was observed that this training method allows for synchronous non-selective muscle fiber recruitment by increasing the rate of firing frequency, resulting in a greater motor unit activation [12] and an improvement in the skeletal muscle’s physiological response and neuromuscular adaptability [13]. Several studies demonstrated the benefits of WB-EMS on muscular strength in moderately trained young adults [14], post-menopausal women [15], obese women [16], athletes [17] and patients with neurological disorders [18]. However, the simultaneous stimulation of large muscle groups combined with high-frequency electrical stimulation can excessively elevate the creatine kinase (CK) levels and potentially lead to rhabdomyolysis [19], increasing the risk of acute renal failure [20] and muscular and cardiac problems [21]. Several studies [22,23] demonstrated a significant increase in CK levels, up to a thousand-fold (240,000 U/L) compared to the normal levels (<180 U/L). The CK levels tend to rise significantly 48–96 h after the end of a training session [24]. Guidelines have been developed for a safe administration of the training protocol [24]. Consequently, it becomes essential, when aiming to suggest a sequence of strength protocols implemented at different levels of intensity with WB-EMS, to assess the most suitable training type in terms of intensity, volume and exercise type and to properly apply additional WB-EMS stimulation. 

The aim of this study was to assess the acute effects of a single session of WB-EMS superimposed over classic resistance training on isometric and endurance strength and flexibility and whether this training method could be adapted to a traditional resistance training protocol for a sample of active women. In addition, this study aimed to evaluate the safety of the protocol in terms of WB-EMS training intensity, evaluating the levels of CK after 48 h. To our knowledge, this is the first study that applies WB-EMS to resistance training exercises following the modalities of classic resistance training in terms of volume, intensity and rest in active women. 

## 2. Materials and Methods

### 2.1. Study Design

This study was designed to evaluate the acute effect of a single session of WB-EMS, using the MIHA-bodytec^®^ device (GmbH, Augsburg, Germany), superimposed over classic resistance training, on isometric and endurance strength and on flexibility in active, fit women, in comparison to traditional resistance training. In addition, the CK levels were analyzed 48 h after the completion of the experimental procedure to assess the safety of the training protocol.

### 2.2. Participants

Sixteen female subjects (aged 22.06 ± 1.88 years) were recruited from the University Sports Centre of Molise (CUS Molise) and were randomly assigned to an experimental group (EG) (*n* = 8) and to a control group (CG) (*n* = 8). The participants had at least 2 years of experience with resistance training and reported a training frequency of 2 days per week. In addition, this was their first experience with WB-EMS training. The sample characteristics are shown in Table 1.

The inclusion criteria were (1) age from 20 to 30; (2) female gender; (3) having performed exercise for fitness for almost 2 years. The exclusion criteria were (1) injuries occurred in the previous 6 months; (2) adherence to other training protocols; (3) use of drugs or medications that could influence the test results and the training exercises; (4) contraindication for the utilization of WB-EMS.

All participants were informed about the study procedures and signed an informed consent. The study was designed and conducted in accordance with the Declaration of Helsinki and approved. The study was conducted at the University of Molise and was approved by the local bioethical committee (11487/2020).

### 2.3. Experimental Procedures

**Figure 1:**
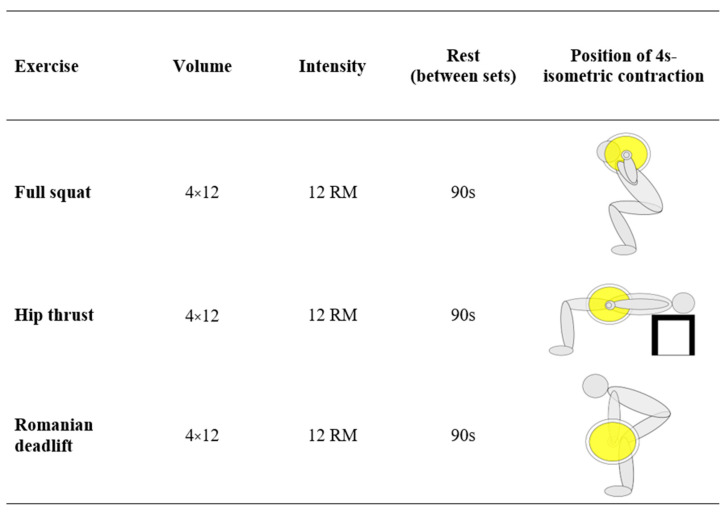
The figure shows the training load of the three exercises performed. In addition, the phase of the exercise where the participants were asked to perform 4 s of maximal isometric contraction with (EG) or without (CG) the superimposition of WB-EMS is highlighted.

The participants were randomized into an EG and a CG by assigning them a progressive number from 1 to 16, with no repetitions. The generation of a random number list was performed using an online software (https://www.random.org/sequences/ (accessed on 17 June 2023), Dublin, Ireland). The list was used to allocate the participants in a random order to the different groups in blocks of two participants per group, following the sequence EG–CG. After randomization, the homogeneity of the two groups was evaluated based on the assessed outcomes. A familiarization session was performed one week before the intervention to make the participants accustomed to the electric stimuli. Both EG and CG training protocols consisted of 3 voluntary strength exercises: full squat, hip thrust and Romanian deadlift. The participants were asked to calculate their 12RM the week before the intervention, supervised by a specialized trainer. After 10 min of warm-up, each exercise was performed in 4 sets with 12 repetitions, with 90 s of rest between the sets and 2 min of rest between the exercises [25]. During each repetition, 4 s of stimulation occurred during the isometric contraction at the point of transition between the eccentric and the concentric phases of the movement; the following 4 s without stimulation were used to return to the initial position (2 s) and then reach the isometric position again (2 s) for the subsequent 4 s of stimulation. The 4 s of stimulation during the isometric phase were programmed to coincide with specific positions during each exercise. The impulse was administered at the point of maximum squatting during the squat, at the point of maximum hip extension during the hip thrust and at the point of maximum hip flexion during the Romanian deadlift. This approach aimed to maximize muscle activation in specific phases of the movements. The training protocol is shown in Figure 1. The EG performed the protocol with the superimposition of WB-EMS (rectangular stimulation at 85 Hz, 350 µs, 4 s stimulation/4 s rest) [26]. The participants were encouraged to exert a maximum effort during the WB-EMS impulse. To ensure an optimal recovery phase, the stimulation was paused during the recovery periods between sets and exercises [26]. However, the effective duration of the training was planned, in relation to exercise volume and intensity, to reach a total of 20 min of stimulation (including both “on” and “off” periods), avoiding the risk of developing contraindications associated with prolonged stimulation. To generate a sufficient but tolerable intensity, the stimulus was set to reach a level of 6–7 out of a maximum of 10 on the Borg CR10 scale measuring the rate of perceived exertion (RPE) [27]. The control group followed the same training protocol without the superimposition of WB-EMS.

### 2.4. Testing Procedures

The participants were asked not to exercise at all during the preceding 48 h.

Isometric and endurance strength and flexibility performance were assessed pre- and immediately post-intervention. Blood samples were collected 48 h after completing the training protocol to assess CK concentration.

#### 2.4.1. Isometric and Endurance Strength

Isometric strength was assessed using the portable dynamometer Suiff Pro S2 (200 kg) (Suiff, Barcelona, Spain). A total of three isometric strength tests were performed: knee extensor and flexors and posterior kinetic chain extensors. The average and peak force generated during the tests were taken into consideration. The isometric strength of the dominant (DL) and non-dominant (NDL) leg was evaluated individually for the knee extensor and flexors. The DL was assessed through the balance recovery test [28]. The participant was gently pushed from behind between the scapulae by the examiner, causing a slight loss of balance. To regain balance, the participant took a step forward. The limb used to regain balance was considered as the DL. For the evaluation of the isometric strength of the knee extensor and flexors, the participants were placed in a seated position, with their hands on the sides of the seat to stabilize themselves and their knees and hips flexed to 90° according to Bohannon [29]. The participants were asked to extend/flex one knee at a time as strongly as possible. For the measurement of the isometric strength of the posterior kinetic chain extensors, the isometric midthigh pull test (IMPT) was used. An immovable bar was placed immediately superior to the patella, with the participants in a standing position [30]. A slight pre-tension before the pull was required to reduce the risk of vertical accelerations for the athlete. The participants were asked to pull against the bar exerting maximal effort. Each trial in each test lasted for 5 s. Three minutes of rest were allowed between each trial to ensure complete muscle recovery [25]. The average and peak isometric force generated during the tests were taken into consideration. For each test, the best value of three attempts was used for the analysis [31].

Isometric endurance was evaluated though the wall sit test. The participants were asked to sit unsupported against a wall, with their knees and hip flexed at an angle of 90°, and to maintain the position as long as possible [32]. The total time, in seconds, spent in isometric position was considered as the outcome for the analysis.

#### 2.4.2. Flexibility

The sit and reach test was used to evaluate the flexibility of the posterior kinetic chain [33]. A box (Acuflex I flexibility tester, height: 30.5 cm) with a centimeter scale placed on its upper surface was used for this assessment. The participants had to sit on the ground with knees extended and feet together, placing the soles of the shoes against the end of the box. Keeping their hands overlapped with the palms facing downward, the participants were asked to slowly bend forward along the centimeter scale, reaching as far as possible without bending the knees. The best of three attempts was considered for the analysis.

#### 2.4.3. Creatine Kinase Activity Measurement

Blood samples were collected under non-fasting condition from an antecubital vein 48 h after training. The blood samples were analyzed in triplicate, and %CV was less than 0.5. Plasma aliquots were stored at −80 °C until the samples were processed. The quantitative measurement of CK activity was carried out using the Ortho Vitros4600 (Ortho-Clinical Diagnostics, Rochester, NY, USA) with the dry chemistry-based CK method (Ortho-Clinical Diagnostics) calibrated against the WHO standard, according to manufacturers’ instructions. The concentration is expressed as unit per liter (U/L).

### 2.5. Power Analysis

Sample size was calculated using G*Power (version 3.1.9.7; written by Franz Faul, University of Kiel, Kiel, Germany). The following design specifications were considered: test family = F tests; statistical test = repeated measures analysis of variance (ANOVA) between factors; α = 0.05; (1 − β) = 0.95; effect size f = 0.5; number of groups = 2; number of measurements = 2. Sample size estimation indicated that 16 participants were required, with a critical F value of 4.600.

### 2.6. Statistical Analysis

Data analysis was performed using SPSS Statistics 21 (IBM) software. The normal distribution of the continuous variables was verified using the Shapiro–Wilk test. The data are presented as mean with standard deviation. Analysis of variance for repeated measures (RM-ANOVA) was used to evaluate differences in physical performance (isometric strength and flexibility), considered as the dependent variable, between the groups (EG and CG), considered as the independent variables. When significant differences in relation to time (pre-test vs. post-test), between the groups (EG vs. CG) and/or in the time*group interaction were found, Bonferroni post-hoc correction for multiple comparisons was applied.

In addition, one-way ANOVA was used to evaluate the differences in the CK levels, considered as the dependent variable, between the groups (EC and CG), considered as the independent variable. Finally, the partial eta square (η^2^p) was calculated to determine the effect size of the analysis. The alpha test level for statistical significance for all variables was set at 0.05.

## 3. Results

RM-ANOVA performed on the DL knee extensors’ mean value showed a significant difference between the groups (F_(1,14)_ = 6.501; *p* = 0.023; η^2^p = 0.317), with the experimental group achieving higher strength mean values than the control group. No significant differences were found for time (F_(1,14)_ = 1.560; *p* = 0.232; η^2^p = 0.100) and time*group interaction (F_(1,14)_ = 1.023; *p* = 0.329; η^2^p = 0.068).

RM-ANOVA performed on DL knee extensors’ peak value showed a significant time*group interaction (F_(1,14)_ = 5.727; *p* = 0.031; η^2^p = 0.290), with the experimental group demonstrating a slight improvement in performance, and the control group experiencing a decline in performance. No significant differences were found for time (F_(1,14)_ = 0.995; *p* = 0.335; η^2^p = 0.066) and between the groups (F_(1,14)_ = 2.473; *p* = 0.138; η^2^p = 0.150).

RM-ANOVA performed on DL knee flexors’ mean value showed a significant time*group interaction (F_(1,14)_ = 7.616; *p* = 0.015; η^2^p = 0.352), with the experimental group demonstrating a slight improvement in performance, and the control group experiencing a performance decline. A significant difference between groups (F_(1,14)_ = 5.531; *p* = 0.034; η^2^p = 0.283) was found, with the experimental group achieving higher strength mean values than the control group. No significant differences were found for time (F_(1,14)_ = 1.031; *p* = 0.327; η^2^p = 0.069).

RM-ANOVA performed on DL knee flexors’ peak performance showed a significant time*group interaction (F_(1,14)_ = 19.351; *p* = 0.001; η^2^p = 0.580), with the experimental group demonstrating a slight improvement in performance, and the control group experiencing a performance decline. No significant differences were found for time (F_(1,14)_ = 0.172; *p* = 0.684; η^2^p = 0.012) and the between groups (F_(1,14)_ = 2.311; *p* = 0.151; η^2^p = 0.142).

RM-ANOVA performed on the posterior kinetic chain extensors’ mean and peak measurements showed a significant effect of time (F_(1,14)_ = 7.932; *p* = 0.014; η^2^p = 0.362 and F_(1,14)_ = 7.294; *p* = 0.017; η^2^p = 0.343, respectively), with the experimental group significantly increasing the strength performance compared to the pre-assessment measurement (*p* = 0.001 and *p* < 0.001, respectively). In addition, a significant time*group interaction was found (F_(1,14)_ = 10.036; *p* = 0.007; η^2^p = 0.418 and F_(1,14)_ = 20.719; *p* < 0.001; η^2^p = 0.597, respectively). No significant differences were found between the groups (F_(1,14)_ = 3.769; *p* = 0.056; η^2^p = 0.236 and F_(1,14)_ = 1.488; *p* = 0.243; η^2^p = 0.096, respectively).

RM-ANOVA performed on the sit and reach test showed a significant effect of time (F_(1,14)_ = 7.932; *p* = 0.014; η^2^p = 0.357), with the experimental group significantly increasing the flexibility performance compared to the pre-assessment measurement (*p* = 0.001). In addition, a significant time*group interaction (F_(1,14)_ = 10.362; *p* = 0.006; η^2^p = 0.425) was found, with the experimental group demonstrating a slight improvement in performance, and the control group experiencing a performance decline. No significant differences were found between the groups (F_(1,14)_ = 0.071; *p* = 0.793; η^2^p = 0.005).

**Figure 2:**
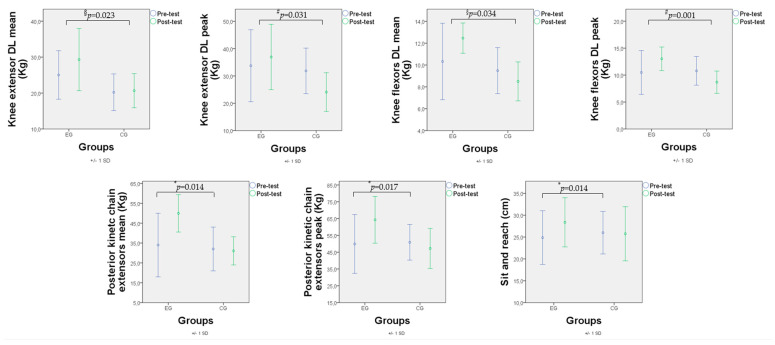
Pre- and post-test means and SD of the results of the performance tests for the two groups. * = pre- and post-test differences (time factor); # = significant time*group interaction; § = significant differences between the two groups (group factor). EG: experimental group; CG: control group; Kg: kilograms; cm: centimeters.

No significant differences were found for NDL knee extensors and knee flexors’ mean and peak measurements and in the Wall sit 90° test (all *p*s > 0.05). All the results are shown in Table 2 and Figure 2.

Finally, ANOVA performed on the CK levels showed no significant difference between the groups (F_(1,14)_ = 0.715; *p* = 0.412; η^2^p = 0.049). The results are shown in Table 3.

## 4. Discussion

The main result of the administration of this WB-EMS protocol was a significant increase in both mean and peak isometric strength of the posterior kinetic chain extensors, assessed by the IMPT test. Significant differences between the experimental and control groups in the mean strength of the knee extensors and flexors were found.

Considering that resistance training acute effects are mainly due to neural adaptations, the WB-EMS application may have induced a mechanism responsible for post-tetanic potentiation (PTP), guarantying an enhanced strength output [34]. PTP occurs when potentiation is induced exogenously by high-frequency electrical stimulation (70–100 Hz) [35]. Requena et al. [36] hypothesized that PTP may induce greater potentiation than post-activation potentiation (PAP), which is induced endogenously by a maximal or near-maximal voluntary contraction [35]. Previous studies demonstrated that even a single session of WB-EMS training significantly increased both upper and lower limb strength [14,37]. Sadeghipour et al. [38] showed significant improvement in leg press maximal strength performance after 6 weeks of WB-EMS in trained women; however, the authors did not show significant differences compared with a strength training group. Dörmann et al. [39] evaluated the short-term effect of strength training with and without the superimposition of WB-EMS on strength and power performances in physically active females. The authors showed significant improvement in strength and power performance in leg extension and leg curl, but no differences between the experimental and the control group were found. Moreover, an increase in strength values in the mid-pull test appeared to correlate with a lower rate of injuries in female athletes [40].

The CK levels 48 h after the completion of the training protocol remained essentially unchanged, confirming the safety of the integrated protocol including electromyostimulation. Despite the average CK levels in the experimental group being twice as high as those in the control group, the levels remained within the physiological range (<180 U/L). No significant differences in CK levels with respect to the control group were found; so, this training protocol did not show contraindications for this population.

A significant increase in both mean and peak isometric strength for both knee extensors and flexors was found only in the dominant limb, while the non-dominant limb did not show significant changes. We hypothesize that subjects involuntarily place more load on their dominant limb, inducing a greater PTP in the DL, with a subsequent enhancement in strength performance. Since WB-EMS increases the stimulus intensity, especially in terms of discharge frequency, it is possible that this protocol induced great postural adjustments in the participants [41], even if it was demonstrated that bilateral resistance training did not reduce the between-limbs asymmetry [42]. The level of fatigue could also accentuate the difference between the dominant and the non-dominant limb, influencing the isometric strength output [43,44].

No significant change was observed in the isometric endurance strength assessed through the wall sit 90° test. This was an expected result, as the test outcome is also influenced by the subject’s motivation and its ability to counteract fatigue. Flemington et al. [45] suggested that the combination of internal focus and mental fatigue can have negative effects on physical endurance performance. A previous study supported a relationship between the socio-cognitive sphere and perceived effort [46], highlighting how having an associative focus, which means being focused on physical effort or on the execution of a high-intensity test, can promote negative thoughts and increase fatigue. Generally, fatigue perception tends to be higher in women compared to men, even when experiencing the same workout intensity [47]. In contrast, several studies indicated that having a dissociative focus, which means not being focused on physical effort or on the execution of a high-intensity test, can promote adherence and tolerance to physical effort [48,49].

Significant improvement was observed in the flexibility of the posterior kinetic chain only after the experimental protocol was completed. Flexibility in women is important for postural stability and balance in daily activities, reducing the risk of osteoarticular injuries and back pain [50]. Several studies showed that resistance training can improve flexibility in women [51,52] by modifying the architecture and structure of the connective tissue, reducing passive tension and tissue stiffness surrounding the joints [53]. The protocols proposed in this study combined a sequence of controlled concentric, eccentric and isometric phases. Several studies showed that combining eccentric and concentric exercise with isometric training can lead to enhancements in muscle function, flexibility and stiffness [54,55]. Since both groups performed the same training exercises, we can hypothesize that the addition of WB-EMS was crucial for increasing flexibility in the experimental group. This result was confirmed by the study of Vaculíková et al. [56] in elderly women.

Several limitations of this study may be addressed: the assessment was conducted using a single test (IMPT), which is specific only to the Romanian deadlift. Electromyography was not performed to evaluate muscle neural activation during experimental training and/or tests administration. While the changes in CK levels were not monitored at various points in time (24 h, 72 h, 96 h), the absence of a concentration increase beyond physiological levels after 48 h suggests that they did not reach critical levels in the subsequent hours.

## 5. Conclusions

This study aimed to assess the effectiveness of a single training session combining WB-EMS with a resistance training protocol. Given the obtained positive acute effects on strength and flexibility performances, we hypothesize that the chronic application of this protocol may lead to further and lasting improvements.

The application of EMS may have played a role in PTP, with high-frequency stimulation superimposed on voluntary isometric contraction resulting in enhanced strength production. Probably, the improvements achieved are not attributable to the electrical stimulus itself but were due to its effectiveness in terms of muscle activation, which likely contributed to the enhanced performance in the analyzed assessments.

Finally, since the CK levels remained within the physiological range, we can conclude that the protocol can be considered safe.

WB-EMS training represents a strategy for acute strength improvement and flexibility enhancement. These improvements were evident even when employing sub-maximal loads (12RM), highlighting the safety of this resistance training approach. This implies that the protocol may be suitable for female athletes with limited experience or inadequate technique, allowing for strength gains without the need for maximal loads. Furthermore, the application of technology provides a diverse range of training possibilities, ensuring motivation and avoiding dropouts [57,58].

## Figures and Tables

**Table 1:** Sample characteristics.

Variable	Mean ± SD
**Experimental group (*n* = 8)**	
Age	22.37 ± 2.20
Training experience (years)	2.75 ± 0.46
Training volume (h/week)	4.88 ± 0.83
**Control group (*n* = 8)**	
Age	21.75 ± 1.58
Training experience (years)	2.64 ± 0.5
Training volume (h/week)	4.81 ± 0.70

**Table 2:** Isometric and endurance strength and flexibility results.

Variable	Experimental Group		Control Group		Time F_(1,14)_	Interaction F_(1,14)_	Intervention Group F_(1,14)_
	Pre	Post	Pre	Post			
Knee extensors DL mean (Kg)	25.04 ± 6.74	29.31 ± 8.65	20.21 ± 5.10	20.66 ± 4.75	F = 1.560 *p* = 0.232	F = 1.023 *p* = 0.329	F = 6.501 *p* = 0.023 *
Knee extensors DL peak (Kg)	33.75 ± 13.18	36.95 ± 11.95	31.86 ± 8.34	24.08 ± 7.08	F = 0.995 *p* = 0.335	F = 5.727 *p* = 0.031 *	F = 2.473 *p* = 0.138
Knee extensors NDL mean (Kg)	29.17 ± 12.47	28.57 ± 7.27	23.62 ± 6.54	24.96 ± 5.47	F = 0.025 *p* = 0.877	F = 0.171 *p* = 0.686	F = 1.739 *p* = 0.208
Knee extensors NDL peak (Kg)	36.20 ± 14.37	36.75 ± 15.77	29.94 ± 8.80	23.21 ± 7.01	F = 1.113 *p* = 0.309	F = 1.545 *p* = 0.234	F = 3.525 *p* = 0.081
Knee flexors DL mean (Kg)	10.32 ± 3.50	12.46 ± 1.38	9.49 ± 2.11	8.50 ± 1.78	F = 1.031 *p* = 0.327	F = 7.616 *p* = 0.015 *	F = 5.531 *p* = 0.034 *
Knee flexors DL peak (Kg)	10.49 ± 4.06	13.02 ± 2.19	10.79 ± 2.66	8.69 ± 2.07	F = 0.172 *p* = 0.684	F = 19.351 *p* = 0.001 *	F = 2.311 *p* = 0.151
Knee flexors NDL mean (Kg)	9.24 ± 2.58	9.09 ± 1.15	8.06 ± 2.22	7.77 ± 0.99	F = 0.101 *p* = 0.756	F = 0.010 *p* = 0.922	F = 3.950 *p* = 0.067
Knee flexors NDL peak (Kg)	12.91 ± 2.34	11.60 ± 1.78	12.40 ± 1.41	11.74 ± 1.30	F = 2.952 *p* = 0.108	F = 0.320 *p* = 0.581	F = 0.080 *p* = 0.782
Posterior kinetic chain extensors mean (Kg)	33.97 ± 16.01	49.94 ± 9.38	32.00 ± 11.02	31.06 ± 7.09	F = 7.932 *p* = 0.014 *	F = 10.036 *p* = 0.007 *	F = 3.769 *p* = 0.056
Posterior kinetic chain extensors peak (Kg)	49.90 ± 17.51	64.25 ± 13.88	50.89 ± 10.60	47.22 ± 11.94	F = 7.294 *p* = 0.017 *	F = 20.719 *p* < 0.001 *	F = 1.488 *p* = 0.243
Wall sit 90° (s)	91.20 ± 29.35	91.62 ± 21.61	83.79 ± 14.74	81.69 ± 9.13	F = 0.031 *p* = 0.862	F = 0.072 *p* = 0.793	F = 0.946 *p* = 0.347
Sit and reach (cm)	24.87 ± 6.15	28.37 ± 5.63	26.00 ± 4.87	25.75 ± 6.19	F = 7.783 *p* = 0.014 *	F = 10.362 *p* = 0.006 *	F = 0.071 *p* = 0.793

DL = dominant leg; NDL = non-dominant leg; * = significant differences. Time: denotes differences between pre- and post-test values (time factor). Interaction: denotes a significant time*group interaction. Intervention group: denotes significant differences between the 2 groups (group factor).

**Table 3:** Creatine kinase levels after 48 h.

Variable	Experimental Group	Control Group	*p*-Value F_(1,14)_
CK levels (U/L)	162.37 ± 232.63	89.25 ± 75.86	F = 0.715 *p* = 0.412

CK = creatine kinase levels; U/L = unit per liter.

## Data Availability

Data are available upon request to the corresponding author.
